# Special Issue “Functional Role of Cytokines in Cancer and Chronic Inflammation”

**DOI:** 10.3390/ijms26094048

**Published:** 2025-04-25

**Authors:** Daria Potashnikova, Elizaveta Fasler-Kan

**Affiliations:** 1 Department of Cell Biology and Histology, School of Biology, Lomonosov Moscow State University, 119234 Moscow, Russia; 2 I.V. Davydovsky City Clinical Hospital, Moscow Department of Healthcare, 109240 Moscow, Russia; 3 Department of Pediatric Surgery, Children’s Hospital, Inselspital Bern, CH-3010 Bern, Switzerland; 4 Department of Biomedical Research, University of Bern, CH-3010 Bern, Switzerland

## 1. Introduction

Cytokines are a diverse group of signaling proteins that are secreted by a wide range of cell types, including, but not limited to, immune cells [1,2,3]. Over 100 cytokines have been identified and their roles in maintaining homeostasis and host defense have been extensively characterized [4]. Through signaling pathways, cytokines contribute to various aspects of inflammation such as cell damage, metabolic alterations, angiogenesis, cell chemoattraction, and migration [5,6].

Beyond their more apparent roles in acute inflammation, these molecules are responsible for a longstanding and diverse chronic inflammatory response that is now known to drive several diseases, such as mucosa-associated gastrointestinal diseases and allergies [7,8,9], chronic respiratory diseases [10,11], neurodegenerative disorders [12,13], and autoimmune diseases [14,15], etc. Special attention is paid to the mediators of chronic inflammation in cancer. Over the past 30 years, the roles of cytokines and their receptors have been thoroughly investigated in relation to both cancer progression and anti-cancer therapy. Cytokines show great promise for therapeutic intervention, as these molecules, including interferons, interleukins, tumor necrosis factors, chemokines, and various growth factors (TGF-β, VEGF, EGF, and many others), can either promote or inhibit tumor growth and thus influence prognosis [16,17,18,19]. However, providing more efficient prognostic and immunotherapeutic approaches to cancer will require studies on tumor heterogeneity and the comprehensive cytokine profiling of each tumor type from their vast diversity. It also becomes evident that cytokines are the key components involved in the pathogenesis of cardiovascular diseases (CVDs) due to their contribution to the inflammatory processes [20,21]. Considering that cardiovascular diseases have the highest mortality and morbidity rates worldwide, this issue arouses much interest. However, it remains to be elucidated which cytokines may be used as disease markers or predictors, and which may be efficiently targeted in CVD prevention and treatment.

This research topic is, thus, dedicated to the new data and the insights concerning various chronic inflammation models—the cytokine signaling patterns observed in these cases persist for years, affect the cross-talks between different cell types, and form a microenvironment that can either promote or block disease progression. Although the Special Issue does not fully capture the breadth of the problem, we are happy to have received manuscripts on prominent disease models, such as cancers, tuberculosis, and cardiovascular diseases. The articles published in the Special Issue cover aspects of both fundamental and translational cytokine research and employ a variety of approaches, including in vitro, ex vivo, and in vivo models. Together, these findings contribute to a better understanding of the role of cytokine signaling in chronic disease pathogenesis and progression. Understanding these processes is important for the further development of new cytokine-specific therapeutic approaches.

## 2. An Overview of Published Articles

### 2.1. Cytokines in Cancer

The cytokine-driven cross-talk between the tumor and tumor microenvironment was investigated by Bogdanova et al. (contribution 1). The authors used a colon adenocarcinoma/cancer-associated fibroblast model to prove that the pro-tumorigenic phenotype of fibroblasts was induced by tumor-secreted factors and similarly reproduced it by TNF stimulation. They attributed the pro-tumorigenic activity of the cancer-associated fibroblasts (CAFs) to the production of IL-6, a pro-inflammatory molecule that enhances the motility of tumor cells and may thus promote metastasis in vivo. Of particular interest was the finding that the two therapeutic regimens had differential effects on IL-6 production by CAFs and tumor cell motility. Only navitoclax (the bcl-2 family inhibitor) reduced IL-6 secretion and suppressed tumor cell motility, thus disrupting the positive feedback loop between tumor cells and pro-tumorigenic stroma.

The work by Fasler-Kan et al. (contribution 2) analyzed cytokine signaling in pediatric kidney tumor cell lines. The authors have shown for the first time that various STAT (signal transducers and activators of transcription) proteins can be activated in these cells by several cytokines including IFN-α, IFN-γ, IL-6 and IL-4. The nuclear translocation of STAT proteins was confirmed by using phospho-specific anti-STAT antibodies, which recognize only activated/phosphorylated STAT molecules. Both IFN-α and IFN-γ caused the upregulation of major histocompatibility complex (MHC) class I proteins, but not MHC class II proteins. Pediatric kidney tumor cell lines also exhibited functional expression of another cytokine signaling pathway. Tumor necrosis factor (TNF-α)-mediated activation of nuclear factor kappa B (NF-κB). The authors assumed that the human pediatric kidney tumor cell lines could be used as in vitro models for profiling the signaling pathways in different cancers.

### 2.2. Cytokines in Cardiovascular Inflammation

In cardiovascular diseases, cytokines are an important part of the pro-atherogenic environment and thus appear to be potential targets for atherosclerosis prevention and treatment. Atherosclerotic plaques are formed in the blood vessel walls and represent sites of chronic inflammation. Potashnikova et al. (contribution 3) compared the systemic and local cytokine profiles in the blood plasma and the ex vivo plaque explants of patients with atherosclerosis. The authors developed and tested a method of xMAP data normalization suitable for cytokine evaluation in different types of biological material and proved that differential cytokine patterns were produced by the plaque in situ and present in the blood flow. The authors stated that plaque-specific cytokines might play a key role in driving immune cell activation and infiltration into atherosclerotic plaques. For several putative cytokines involved in immune cell infiltration (CCL2, CCL3, CCL4, CCL5, and CX3CL1), the cellular sources were determined. Thus, it was shown that different cell populations in the plaque contributed differently to its immune profile.

Savchenko et al. (contribution 4) demonstrated that bioactive peptide galanin plays a critical role in regulating cardiovascular homeostasis. The authors found that galanin coordinated fibro-inflammatory responses and mitochondrial function in post-infarction reperfusion injury. The cardioprotective effects of galanin in attenuating myocardial reperfusion injury were associated with the promotion of mitochondrial biogenesis and preservation of mitochondrial integrity. In post-infarction tissue remodeling, galanin regulated the release of inflammatory cytokines and infiltration of inflammatory cells. It was shown that galanin receptors were upregulated by the transcription factor nuclear factor-κB (NF-κB), which is increasingly recognized as critical in the pathophysiology of inflammatory diseases.

Kang et al. (contribution 5) focused on age-related alterations in cardiac function. The authors examined age-specific cardiac and metabolic phenotypes in relation to the inflammatory status and antioxidant capacity in mice. The results of their study demonstrated that middle-aged animals exhibited an altered metabolic profile compared to young mice, whereas the myocardial expression of pro-inflammatory cytokines such as TNF-α, IL-1β, IL-6, and IL-10 remained unchanged. In contrast, older animals exhibited increased expression of pro-inflammatory cytokines. These findings highlight the role of several cytokines in age-related events of cardiac dysfunction and may have important implications for the treatment of heart diseases in aging. Age-associated cardiac phenotypes in murine models reflect age-related functional and metabolic alterations in humans.

The interrelationships between living organisms and their environments are complex and interdependent. Several studies suggested that prolonged exposure to electromagnetic fields can have biological effects on body tissues and cells. Savchenko et al. (contribution 6) examined the metabolic, apoptotic, and fibro-inflammatory profiles of the cardiac tissue exposed to a prolonged (14 days long) exposure to 900 MHz environmental electromagnetic fields in a mouse model. The authors showed that the tissue structure and apoptotic, antioxidant, metabolic, and fibro-inflammatory profiles of the heart remained stable under these conditions. An analysis of fibro-inflammatory cytokines including TGF-β1, IL-6, and CCL2, showed unchanged myocardial and inflammatory status in the myocardium exposed to electromagnetic stress for 14 days. It is crucial to continue scientific research in this area to better understand the potential effects of electromagnetic fields on living systems.

### 2.3. Cytokines in Tuberculosis

Infectious diseases, such as tuberculosis (TB), may also develop over many years and form chronic inflammatory patterns within infected tissue. The impressive variation in clinical courses and prognoses for pulmonary TB may imply that several varying patterns exist in the population. Pavlova et al. (contribution 7) evaluated the gene expression signatures of pulmonary TB and associated them with inflammatory activity and *ABCB1* gene expression. The *ABCB1* gene, encoding P-glycoprotein (P-gp), plays a crucial role in regulating inflammation and reducing chemotherapy efficacy, thereby influencing the pharmacokinetics of anti-TB drugs. In lung tissue specimens from patients with TB, high *ABCB1* expression was correlated with increased expression of *HIF1a*, *TGM2*, *IL-6*, *SOCS3*, and *STAT3*. Conversely, a separate pro-inflammatory signature showed elevated expression of *TNF-α* and *CD163* along with low *ABCB1* expression. These findings highlighted the heterogeneity of the inflammatory mechanisms in TB and offered valuable insights into the potential of host-directed therapies to enhance treatment outcomes.

### 2.4. Vitamin B12 and Cytokine Production

Contribution 8 is a review provided by Simonenko et al. and focused on the role of micronutrients, namely vitamin B12, in modulating cytokine levels and the possible onset or progression of diseases related to chronic inflammation. The authors conducted an in-depth literature analysis on vitamin B12 transport, protein partners, mechanisms of action, and consequences of its deficiency at the cellular, tissue, and organism levels. Vitamin B12 is presented as a modulator of cell senescence and the respective secretory phenotype. Of particular interest is the interconnection of vitamin B12 levels with IL-6 production that drives the pro-inflammatory responses in many other instances. The visualization provided in this review is very helpful for understanding the topic.

## 3. Conclusions

This research topic, thus, includes seven diverse original research papers and one review. The articles cover different aspects of cytokine signaling in the models associated with chronic inflammation. The articles support the concept of complex biochemical networks regulating cellular interactions. Together, these papers highlight the fact that understanding disease pathogenesis and providing efficient immunotherapies require comprehensive cytokine profiling of each condition to correctly predict the therapeutic impact.

## Data Availability

Not applicable.
